# Adaptive sampling with Oxford Nanopore offers a simple way to improve the efficiency of plant metagenomic studies

**DOI:** 10.1111/nph.70450

**Published:** 2025-08-05

**Authors:** Joost Theo Petra Verhoeven, Aditya Sanjay Malwe, Nathan Roussel, Ida Broman Nielsen, Sarah S. T. Mak, Tue Kjærgaard Nielsen, Christopher James Barnes

**Affiliations:** ^1^ Centre for Evolutionary Hologenomics The Globe Institute, Faculty of Health, University of Copenhagen Oester Farimagsgade 5 Copenhagen 1014 Denmark; ^2^ Department of Agroecology, Faculty of Technical Sciences Aarhus University Forsoegsvej 1 Slagelse 4200 Denmark; ^3^ Department of Plant and Environmental Sciences, Faculty of Science University of Copenhagen Thorvaldsensvej 40 Copenhagen 1871 Denmark

**Keywords:** adaptive sampling, host depletion, long‐read sequencing, maize, metagenomics, microbial enrichment, nanopore sequencing, plant‐microbe interactions

## Disclaimer

The New Phytologist Foundation remains neutral with regard to jurisdictional claims in maps and in any institutional affiliations.

## Introduction

Shotgun metagenomics is prohibitively inefficient to implement when host DNA dominates reads, such as sampling for endophytes. Uniquely, sequencers from Oxford Nanopore Technologies can perform adaptive sampling where target sequences are actively enriched during sequencing. In this Letter, we outline how the efficiency of metagenomic studies can be improved by reversing this process, with very large improvements possible when the host DNA is the majority of reads, with microbial reads rising from 1.6% of total reads to 18.7% from maize roots. Importantly, this process is easy and free to implement when the host reference genome is available.

High‐throughput sequencing has revolutionised microbial ecology. Initially, DNA metabarcoding offered a cost‐effective way of profiling plant microbiomes without culturing (Ficetola & Taberlet, 2023). However, the approach suffers from some major limitations, including the introduction of compositional biases by the PCR process, the taxonomic resolution being limited and the approach providing only limited functional insight into plant microbiomes. Alternatively, shotgun metagenomics has also been performed for many years and overcomes these limitations, but it too has its own set of limitations (Huang *et al*., 2024). For example, to adequately profile the microbiome, much larger quantities of DNA need to be sequenced (millions instead of thousands of reads). This is not a problem when microbial DNA dominates the source material, but often plant samples contain a considerable proportion of host DNA (Khoiri *et al*., 2021). In these instances, many times more total reads need to be produced, with the majority being discarded in order to generate the millions of microbial reads required to adequately profile the plant microbiome. This makes the approach prohibitively expensive and wasteful for many researchers to perform at scale.

To tackle this issue, researchers have developed multiple strategies, including developing specialised protocols, for example extracting DNA from rhizosphere soil instead of roots directly (Quiza *et al*., 2021). However, these approaches risk missing endophytes completely and biasing the community profile towards loosely adhering microbes. Alternatively, methods have been developed to inhibit the sequencing of highly methylated (i.e. the plant) DNA, but these add cost and complexity to experiments (Heravi *et al*., 2020). Ultimately, a cheap and simple method to increase the proportion of microbial reads within metagenomic studies is highly desirable.

Here, we hypothesise that the feature ‘adaptive sampling’ performed by Oxford Nanopore Technologies (ONT, UK) can fulfil this need. Typically, the feature is used to enrich preloaded target sequences. Here, the sequencer analyses reads in real time by mapping them to the reference genome. The decision to accept or reject a read is made after sequencing the first 400 bases (the ‘adaptive sampling chunk’) when the basecaller determines whether this chunk falls within any target region within the reference file (ONT; ADS_S1016_v1_revM_11Dec2024). If a read is rejected, the polarity of the applied potential on the pore is reversed, and the DNA molecule is ejected from the given pore. The method has been used to enrich both plant genes (Belinchon‐Moreno *et al*., 2025) and specific host‐associated microbes (Martin *et al*., 2022). However, this operation can also be inverted so that sequences matching target regions within the references are rejected (depletion mode), but this is less commonly performed. Despite earlier promising results in cows and animals (Marquet *et al*., 2022; Ong *et al*., 2022a,b), this has not been assessed using plants, to the best of our knowledge.

**Fig. 1:**
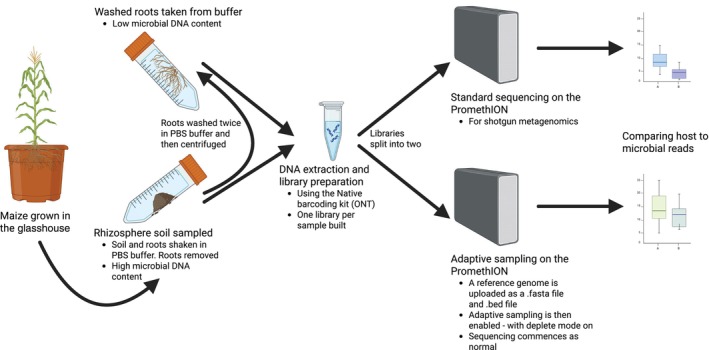
Overview of the project. Maize W22 inbred plants were grown in the glasshouse and sampled after 5 wk. Roots were shaken in phosphate buffered saline (PBS) and the rhizosphere soil collected. Roots were then removed and washed again, with the remaining soil and buffer discarded. Rhizosphere soil and the twice‐washed roots then underwent library building with a Native Barcoding Kit (Oxford Nanopore Technologies, UK). Libraries were split into two and underwent shotgun metagenomics or adaptive depletion on a P2 Solo (ONT). The effect of adaptive depletion was then tested for by comparing the number of reads assigned to the host or the microbiome.

We hypothesised that, using the plant genome as a depletion reference, we can reject host‐derived reads and increase the proportion of the microbial reads within metagenomic studies. We henceforth refer to this process as adaptive depletion to differentiate it from adaptive sampling (target enrichment). To test this, we utilised maize plants (W22 near isogenic line) (Springer *et al*., 2018), partitioned into samples of rhizosphere soil that are expected to have a high proportion of microbial reads and the remaining washed root samples that have a very low proportion of microbial reads (i.e. unlikely to be viable without enrichment) (Fig. 1). We then ran samples on a PromethION P2 Solo (ONT, UK) under standard metagenomic conditions, or with adaptive depletion activated, and compared whether this significantly increases the total proportion of microbial reads in each sample type.

## Materials and Methods

Maize (*Zea mays* subsp. *mays* L. (W22)) were grown in standard potting soil in a glasshouse in Flakkebjerg (Slagelse, Denmark) and kept at *c*. 24°C in 16 h : 8 h, day : night cycles. After 5 wk, the root network was extracted and shook to remove loosely adhering soil. It was cut into *c*. 20 mm lengths and homogenised. Roots under 3 mm in diameter were placed into phosphate buffered saline (PBS) solution (Quiza *et al*., 2021). These were shaken for 25 min at 250 rpm on a horizontal shaker and subsequently centrifuged for 5 min at 2000 ***g*** to pellet the soil. Roots were then transferred into a new tube of PBS, and the process was repeated. Twice‐washed roots were then freeze‐dried overnight alongside the rhizosphere soil from the first wash. DNA was extracted using a QIAGEN Power Soil Pro Kit (Qiagen), with 250 mg of rhizosphere soil and 100 mg of roots used as starting material. DNA extracts underwent size selection, which was performed using Magbio (MagBio Genomics, Gaithersburg, MD, USA) in a 0.4 bead to sample ratio, removing DNA fragments < 2000 bp.

All 20 DNA extracts were then barcoded and built into libraries using the Native Barcoding Kit 24 V14 (SQK‐NBD114.24; Oxford Nanopore Technologies, Oxford, UK) following the manufacturer's guidelines. Sequencing was performed using a single flow cell (R10.4.1; FLO‐PRO114M) on the PromethION 2 Solo (ONT). Initially, standard sequencing was performed before being stopped after 2 h. The flow cell was then washed and the library reloaded with adaptive sampling enabled (with depletion mode activated), using the W22 genome as the reference (GenBank assembly – GCA_001644905.2). The sequencer was then left to run for *c*. 72 h.

Sequencing data were generated using the MinKnow software (v.24.06.10). The resulting Portable Data 5 (POD5) files were basecalled and demultiplexed using Dorado (0.8.2) with duplex reads enabled. Cutadapt (Martin, 2011) and Nanofilt (v.2.8.0) (De Coster *et al*., 2018) were used to remove low‐quality reads (< 1000 bp and low‐quality reads *q* < 15). Minimap2 (v.2.26) was then used to remove the host DNA using the W22 reference (Li, 2018), with the map‐ont flag enabled. Finally, Kraken2 (v.2.1.3) was used to confirm whether reads were microbially derived (Wood *et al*., 2019). Specifically, the nonhost reads were mapped to the standard Kraken2 microbiome database.

## Results and Discussion

**Fig. 2:**
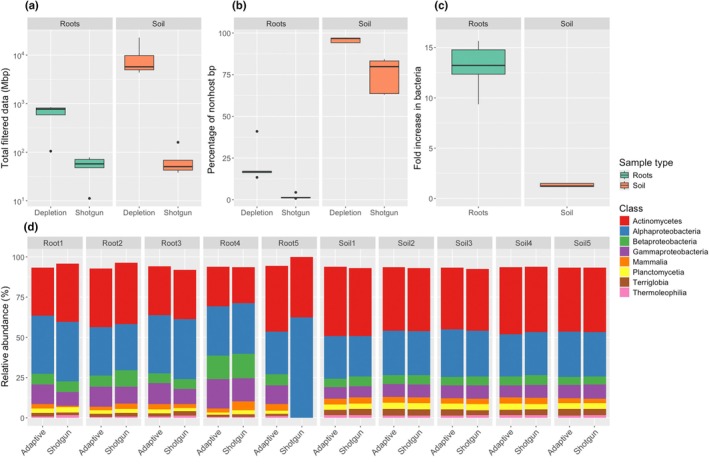
The impact of adaptive sampling on the efficiency of profiling the microbiome of rhizosphere soil and roots of inbred W22 maize was assessed. (a) The total data produced for the shotgun data were considerably less than for the adaptive depletion approach for both the root and soil samples, with an average of 62.5 (±39.2) Mbp produced for the shotgun, but 5084.4 (±6995.3) produced for the adaptive. For the boxplots, the thick line represents the median, while the upper and lower box edges represent the interquartile range and the vertical lines represent the largest/smallest value of the data that is a maximum of 1.5× the interquartile range. Dots represent values that fall outside of this range. (b) Meanwhile, the mean percentage of nonhost reads significantly increased with adaptive depletion in the samples with low (roots) and high (soil) proportions of microbial DNA. This rose from 74.8% (±10.6%) to 95.8% (±1.6%) in the soil, and considerably from 1.8% (±1.5%) to 20.9% (±11.3%) in the roots. (c) Ultimately, this resulted in a fold increase in the reads mapped to bacteria, which rose from a mean average of ×1.3 in the soils and ×13.0 in the roots. (d) The community profile was plotted at the class level and compared between the shotgun and adaptive depletion approaches. The soil samples show consistent results between the two approaches. Meanwhile, the root samples showed more variation, but this is unsurprising given the very low read numbers assigned to the microbiome in these samples. Note that the low abundance classes (< 1% relative abundance) were excluded for visual purposes.

At 74.8% (±10.6%), the percentage of nonplant host reads in rhizosphere soil samples, as produced via shotgun metagenomics, was comparable to other studies (Azevedo‐Silva *et al*., 2021; Quiza *et al*., 2021). Meanwhile, as predicted, washed root samples produced only a very small percentage of nonhost‐derived reads, accounting for just 1.8% (±1.5%) of total reads. Interestingly, this rose significantly for both the rhizosphere soil (*t* = 5.20; *P* = 0.006) and the roots (*t* = 4.33; *P* = 0.012), to 95.8% (±1.6%) and 20.9% (±11.3%) of reads, respectively, with our depletion approach (Fig. 2b). This represented a 1.3‐fold increase for the rhizosphere soil and a 13.1‐fold increase for the roots (Fig. 2c). Of this nonhost data, the majority was assigned to bacteria (88.2%), with only a fraction unassigned (Supporting Information Table S1).

Unfortunately, the shotgun libraries were limited in size, producing a mean of 12 675 (±6688) quality‐filtered reads per sample compared with the 837 569 (±1060 793) reads per sample produced in the adaptive sequencing (Fig. 2a). While not sufficient to profile the entire microbiome, this represents many datapoints for the comparisons between methods. Furthermore, these reads had a mean sequence length of 5033 bp, equivalent to 33 times the length of the standard 150 PE Illumina sequencing, which accounted for 62.5 Mbp (±39.3) data per sample. The rhizosphere soils showed no obvious skew in the taxonomic composition between these approaches, suggesting the adaptive depletion approach is not biasing the community profile in a major way (Fig. 2d). We specifically did not use mitochondrial and chloroplast genomes in the adaptive reference sequences due to their high sequence homology to rhizosphere/root bacteria, as this could lead to more closely related microbes to the plasmids being selected against. Further studies are required to investigate whether specific microbial taxa or regions of microbial genomes are suppressed in the technique. Meanwhile, as only a fraction of reads were nonhost derived from the root samples, this led to very few microbial reads from the shotgun dataset being assigned to bacteria, explaining the variability in the community compositions produced between the shotgun and adaptive depletion approaches (Fig. 2d). Therefore, we performed an additional check to determine whether differences in read depths were biasing our results. This was performed by subsetting the adaptive depletion data to 10 000 reads per sample to be comparable to our shotgun dataset, and then the subset data were reprocessed. The proportion of nonhost reads was significantly higher in the subset data than in the complete dataset (*t* = −8.55; *P* = 0.001) (Table S2), rising from 95.8% to 97.7% being nonhost. However, both of these values deviate much more from the percentage of nonhost reads produced with the shotgun method (74.8%), suggesting the lower read depths in the shotgun metagenomic data was very unlikely to be the main cause of the differences in nonhost reads observed between methods.

While our results suggest the adaptive depletion is powerful, it is not without limitations. First, a reference genome is required, and this is not available for many species (Marks *et al*., 2021). While the approach is likely to be effective across plant species and genotypes, this needs confirmation. More likely, lower genome quality and contiguity will reduce the effectiveness of the host depletion. Related genomes could potentially be substituted, but this too will likely impact the efficiency by which host reads are rejected. Furthermore, adaptive depletion cannot be performed on DNA contaminated by a mixture of hosts, such as bulk soil samples. Second, ONT sequencers continue to have higher error rates than other sequencing platforms (such as PacBio and Illumina) (Sevim *et al*., 2019; Cook *et al*., 2024). Error rates have reduced in recent years to the extent that it was possible to assemble microbial and plant genomes without supplementing with more accurate short‐read data (Sereika *et al*., 2022). For example, reads here had a mean q‐score of 19.7, or *c*. 1% error rate. However, it remains much less accurate and thus requires more sequencing depth than other methods.

## Conclusions

While further testing is needed, these results suggest adaptive depletion is highly beneficial in scenarios in which host genomic data need to be filtered and a reference host genome is available. Importantly, adaptive depletion does not require additional laboratory work, is free and easy to implement and does not seem to bias the community composition. Furthermore, the approach is beneficial at both high and low microbial DNA proportions. Given the ease of adaptive depletion to implement and the lack of adverse effects on the composition, we highly recommend researchers deploy the approach where possible.

## Competing interests

None declared.

## Author contributions

JTPV, ASM and CJB performed bioinformatic analyses. NR, IBN and SM prepared samples and processed them in the laboratory. TKN, IBN and SSTM helped to calibrate equipment and generate data from the sequencer. CJB conceived of the concept. All authors contributed to the initial manuscript draft and to its revisions. JTPV and ASM contributed equally to this work.

## Supporting information


**Table S1** Metagenomics data were produced from maize roots/rhizosphere soil via standard shotgun metagenomics or via adaptive sampling, and the ratio of host and nonhost reads were determined.
**Table S2** Adaptive data from maize roots/rhizosphere soil contained many more reads than the shotgun data; therefore, it was subsampled to a comparable size to the shotgun data and reanalysed for potential biases.
Please note: Wiley is not responsible for the content or functionality of any Supporting Information supplied by the authors. Any queries (other than missing material) should be directed to the *New Phytologist* Central Office.

## Data Availability

All data are freely available on the NCBI under the BioProject accession no. PRJNA1257062.
